# More Competences than You Knew? The Web of Health Competence for European Union Action in Response to the COVID-19 Outbreak

**DOI:** 10.1017/err.2020.35

**Published:** 2020-04-16

**Authors:** Kai P. PURNHAGEN, Anniek DE RUIJTER, Mark L. FLEAR, Tamara K. HERVEY, Alexia HERWIG

**Affiliations:** * Law Group, Wageningen University and Research, Wageningen, The Netherlands; email: kai.purnhagen@wur.nl. On leave to the University of Bayreuth, Faculty of Life Sciences: Food, Nutrition and Health, Campus Kulmbach.; ** Amsterdam Law School, University of Amsterdam and Amsterdam Centre for European Law and Governance, Amsterdam, The Netherlands; email A.deRuijter@uva.nl. De Ruijter acknowledges funding from the Netherlands Organisation for Scientific Research (NWO-veni).; *** School of Law, Queen’s University Belfast, Belfast, UK; email: m.flear@qub.ac.uk. Flear acknowledges funding from the UK Economic and Social Research Council ES/S00730X/1.; **** School of Law, The University of Sheffield, Sheffield, UK; email: T.Hervey@sheffield.ac.uk. Hervey acknowledges funding from the UK Economic and Social Research Council ES/S00730X/1.; ***** Law Group, Wageningen University and Research, Wageningen, The Netherlands; email: alexia.herwig@wur.nl.

## I. Introduction

The European Union (Union) response to COVID-19 has been viewed as underwhelming. Observers are puzzled as to why a threat that seems so similar in severity is evoking such a variety of responses from Union Member States. Further, there is widespread public criticism of the Union for apparently failing to support its own Member States where, for instance, China, Russia and Cuba have done so.^1^ By early April 2020, calls for the Union to undertake debt mutualisation – so-called “coronabonds” – to help pay for recovery had grown stronger, threatening the Union’s very future.^2^

The spectrum of potential measures taken at the Union level is wide. Disaster response regulation regularly comprises risk assessment, advance planning and mitigation, concrete emergency measures and recovery. Our main focus in this short article is on concrete emergency measures, although we also touch on recovery: a fuller analysis would involve more space than is available here.^3^

To combat COVID-19, unlike its Member States, the Union may act “only within the limits of the competences conferred upon it by the Member States in the Treaties to attain the objectives set out therein”.^4^ As legal scholars, we understand why one may think that the Union has no power to act in ways that public health experts and others, such as economists and behavioural psychologists, suggest would be helpful. The Union’s powers in the health domain are traditionally understood to be severely constrained: health law and policy are seen as matters for Member States.

We propose an alternative to this standard legal analysis. The Union has more possible legal powers to create health law and policy in response to the COVID-19 outbreak than is traditionally understood, particularly if the different iterations of the protection and promotion of public and human health throughout the Treaty on the Functioning of the European Union (TFEU) are read *in relation to* one another. This alternative interpretation of the Union’s competence norms, the “legal bases” on which the Union institutions act to adopt either binding legal rules or persuasive measures, suggests that there are legal options that permit the Union a wider range of actions than it has taken to date, and that support – and go further than – the approaches that the European Commission (Commission) and European Centre for Disease Prevention and Control have suggested in various policy documents, guidance and communications in March and April 2020.^5^ In short, we are arguing that *legal* impediments to Union action are less restrictive than is commonly understood.

## II. Specific competence norms: the legal bases for Union law and policy on COVID-19

### 1. Encourage, support, coordinate, resource

The Union enjoys significant powers to encourage, support and coordinate cooperation between the Member States in the area of public health, in general,^6^ and in the context of natural disasters.^7^ Specific competence norms support, for example, cooperation between Member States in cross-border healthcare,^8^ in biomedical and other research^9^ and in civil protection.^10^

The Union has already lawfully taken binding decisions that coordinate actions of the Member States in epidemiological surveillance, and has done so since the 1990s.^11^ The Union has power (eg through coordination decisions taken within the Health Security Committee^12^) to encourage Member State cooperation in adopting passenger screening measures, requiring the temperature of passengers to be taken before boarding a flight.^13^ The Member States took coordinating measures of that nature as an intergovernmental act in 2009 during the swine flu epidemic. But now the formal recognition of the Health Security Committee in Union law suggests that such a coordinating decision could lawfully be taken as a Union act.

The Union also enjoys significant powers to deploy its own (admittedly relatively limited) resources, such as through its structural funding.^14^ The Union has competence to fund collaborative research^15^ into vaccines, treatments, new medical equipment and devices; epidemiological research into the spread of COVID-19; behavioural research into effective disease containment strategies; and social science research into the social, economic, political, legal and cultural consequences of COVID-19. Since 2007, the Union has had competence, under the “solidarity clause”,^16^ if its Member States so desire,^17^ to pool resources under a Civil Protection Mechanism. Since March 2019, the Civil Protection Mechanism has been strengthened by “rescEU”, in an attempt to centralise Union capacities,^18^ permitting the Union to use its internal funds, pre-committed national funds and Union-co-financed Member States’ capacities at the disposal of Union efforts to respond to a major emergency. This gives Union competence, for instance, to organise a European medical corps and a distinctive COVID-19 public procurement scheme.^19^

The Union also has power to permit Member States to deploy resources in response to COVID-19 in ways that distort competition in the internal market;^20^ that is, by giving state aid to key industries.^21^ The Commission has proposed disapplication of the normal “one time, last time” principle,^22^ and it will permit national measures ensuring access to liquidity and finance, preserving employment,^23^ and, crucially, facilitating COVID-19-relevant research and development, supporting the construction and upgrading of testing facilities of COVID-19- relevant products and expanding production capacity for products needed to respond to the outbreak.^24^

### 2. The limits of Union harmonisation

The Union does not have competence to adopt measures that have as their *primary objective* the “harmonisation” of national definitions of health policy; or the organisation and delivery of health services and medical care, including management of health services and medical care and the allocation of resources;^25^ or of national public health laws on tobacco or the abuse of alcohol.^26^ But these constraints on Union competence do not mean “that harmonising measures … cannot have any impact on the protection of human health”.^27^ The Union may lawfully adopt measures that have the effect of improving health, so long as those measures remove obstacles to trade or remove appreciable distortions of competition.^28^ Union internal market measures must protect and promote public health,^29^ and where new scientific evidence comes to light, the Union must update existing harmonised measures.^30^ Thus, the Union may, for example, amend its packaging laws to contain COVID-19 spread through hard surfaces^31^ and protect consumer health.^32^ The Union also has competence to protect the environment,^33^ and so could, for instance, adopt the World Health Organization (WHO) technical standards on water, sanitation, hygiene and waste management for COVID-19^34^ in Union law.

The Union also has competence to *delay* ^35^ and to *relax* harmonised standards from the point of view of individual human health in order to protect population health. Such a relaxation of standards would, for instance, assist in permitting the production of less highly specified personal protective equipment, which offers some, though less, protection against the transmission of COVID-19; or the production of ventilators with three tubes, rather than one. The Union is competent to promote mutual recognition across the internal market of fast-track qualifications of medical professionals, introduced by several Member States in response to COVID-19,^36^ and to reduce the minimum time required to qualify in a medical specialism.^37^ This Union competence is consistent with Union internal market powers, not least because it leaves Member States more discretion to adopt higher domestic standards.

### 3. Union “incentive measures”

The extent of the Union’s powers to adopt binding measures that *incentivise* Member States’ actions “to protect and improve human health and in particular to combat the major cross-border health scourges”^38^ is untested. “Incentivisation” must mean something falling short of legally mandating Member State compliance with a “harmonised” norm. But beyond that, its meaning is unclear.

To “incentivise” means to offer a benefit for or impose a detriment from a particular action: incentivisation can involve “carrots or sticks”. Positive incentives available to the Union to offer to its Member States include access to resources, such as Union funding; collectively held or secured equipment, human resources, pooled expertise or knowledge, administrative capacity or information;^39^ and the Union’s geopolitical capital. Negative incentives include exclusion from those resources, and also fines or other sanctions adopted against Member States.

Incentive measures could be combined with binding Union coordinating measures to enhance national compliance. The Union might thus be empowered to reinforce measures that are already required by secondary Union law, such as reporting through the Union’s Early Warning and Response System (EWRS), including sharing personal data necessary for contact tracing.^40^ Going further, Union law could provide positive incentives to Member States that, for example, impose temporary travel bans; or even those Member States that mandate curfews at the first outbreak sites. Incentives could, for instance, involve access to more Union resources for those Member States that reach certain targets of disease control and containment of COVID-19, encouraging Member States, for example, to impose strict quarantine measures. The Union’s basis for action is stronger in the case of Union measures like these that seek to prevent the outbreak of a communicable disease becoming a *cross-border* problem, or to reduce the cross-border nature of a serious health threat such as COVID-19.

In adopting incentive measures, the Union must respect national responsibilities for health services and medical care.^41^ Measures that would incentivise Member States to adopt particular kinds of treatments or to increase the number of intensive care units or doctors are outside Union competence. Nonetheless, taking into account general principles of Union law, such as the precautionary principle, the proportionality principle and especially the Union’s Charter of Fundamental Rights, Article 35, on the right to healthcare, we might consider the possibility of Union competence to incentivise Member States to provide localised, temporary, ad hoc and context-specific solutions to concrete cross-border problems *with regards to health services* that arise when fighting threats to public health, such as COVID-19. The Union perhaps has competence to adopt measures that, for example, provide resources to health service providers working together on COVID-19 responses across an internal Union border where existing health corridors already involve sharing resources.^42^

Even where there is no direct cross-border element, such as in the case of school closures or curfews, the Union might have competence to incentivise these national measures where there is a serious health threat. Here, we note the European Court of Justice’s (ECJ) approach to interpreting a “cross-border” element in internal market law: even a potential effect on the ability of factors of production (including people) to cross borders is enough to bring the matter within the scope of Union law.^43^

Finally, and most ambitiously, if we consider that a “backstop” of Union measures that apply in the absence of Member State action would “incentivise” Member States to act, the Union would have competence to adopt a very wide set of measures indeed. In the absence of domestic measures to coordinate, the Union might – on an untested interpretation of the relevant competence norm – have the power to adopt binding risk-based, local and temporary measures to contain the spread of COVID-19 that apply in the absence of domestic action. This approach skates very close to “harmonisation”, hence would only justify temporary and localised mandatory Union acts.

## III. The “web of competence”: more than the sum of its parts

The analysis above shows that the competence norms on which Union COVID-19 responses could be based are spread throughout the Union’s enabling Treaties. But what if, instead of treating each competence norm as a thread, or even tightrope, upon which the Union must carefully balance in order to take action, we looked at all of the threads together? Taken together, we argue, the Union Treaties provide a *web* of competence, which is stronger than its individual threads, and as such extends its legal powers beyond those of the separate threads of the discrete competence norms.

This web of competence is based on the following aspects of the Treaties, which together provide the basis for interpreting its competence norms, placing the individual competences or threads in relation to one another and allowing them to be woven into the web. Protection of human health is a general principle or value inherent in Union law, which takes precedence over mere economic considerations.^44^ The Union is obliged to “mainstream” human health in all of its policies and activities.^45^ It has a residual power,^46^ where no specific competence norm is present where necessary to achieve its objectives: this was the basis for Union health law and policy before 1993.^47^ Textual differences between “human health” and “public health”, which appear to distinguish types of Union competence (harmonisation, incentivisation, coordination), do not do so on closer inspection.^48^

Objections to the idea that the Treaties embody a “web of competence” greater than the sum of its separate parts go to questions of legitimacy of the Union as a non-state entity that nonetheless takes decisions on behalf of its people(s). These are well rehearsed in the literature, and we cannot resolve them here. But we note that the Union is far from a “complete” democracy, and that constitutional-type limitations on its powers are even more necessary than limits on the governments and administrations of states. In putting forward the idea of a “web of competence”, we do not mean to argue that the Union has *unlimited* or extra-textual competences. Rather, we propose that the enabling texts of the Treaties be considered as a whole. In some ways, this approach constrains the Union as much as, if not more than, an interpretation that treats the separate competence norms individually.

If accepted, the TFEU’s web of competence would provide a legal basis for a wider range of acts to combat COVID-19 than those discussed so far.

For example, the Union would have competence to keep borders open to essential products in order to protect health.^49^ If needed, the Union’s health competences^50^ could be used as a legal basis to put pressure for compliance on Member States through incentive measures (sanctions or limiting access to data and EU protection mechanisms such as the EWRS). This inverts the traditional legal approach that health protection is an *exception* to the rule of free movement, and thus *Member States* may derogate from internal market rules^51^ where necessary to protect public health. It is the *Member States*, on this interpretation, who can control or interrupt supply chains in the internal market, such as to prevent the export of medical equipment, devices or medicines necessary to combat COVID-19 and vital supplies such as food. The Union institutions only have the power to check for the proportionality of the measures, focusing on the domestic level. Member States can show that measures meet what is required by reference to the specific needs of *their populations*.^52^

The web of competence suggests a different approach to the Union’s competence to control national measures on the basis of proportionality.^53^ This approach is based on the principle of solidarity between Member States,^54^ the Union’s obligation to protect public health^55^ and the right to healthcare within Union law.^56^ On this approach, scarce products needed for combatting COVID-19 need to be “channeled to those who need them most”,^57^ and this means keeping internal Union borders open for the supply of essential products, especially food, as well as medical equipment, devices and medicines. The internal market is at the service of the health of *Europe’s* population. This is a novel interpretation of internal market law, not based on case law or any other legal instrument. It assumes a concept of Union public health and Union solidarity, rather than the usual interpretation where public health is a policy area in which domestic solidarity *within* Member States (and Member States’ sovereignty in the health domain) is the principle that has to be traded-off against market integration.

According to the Commission, this novel interpretation of proportionality means that an outright export ban on essential products will not be deemed proportionate.^58^ National measures need to be aimed at ensuring that the products reach the persons who need them most *considered at a Union level*, and they need to suit the objective of protecting the health of people (again, at a Union level) who need them most. This is not the usual interpretation of Article 35 TFEU on export bans, or Article 36 TFEU, which was never intended to serve public health *at Union level*. But it is an approach that recognises the deeply integrated nature of the single market in goods and the drastic effects that abrupt closure of complex supply chains would have at a time when the Union’s populations are already at risk.^59^ The web of competence suggests that the Union institutions are empowered to take such decisions on this basis, preventing individual Member States from hoarding certain products or limiting their suppliers’ access to the European market.

Further, the web of competence would extend Union power over the movement of people within and into the Union. Again, the power to restrain free movement of people, *inter alia* on public health grounds, is traditionally seen as being reserved to the Member States.^60^ This division of powers suggests that the Union has no competence to act in response to COVID-19 by unified and mandatory restrictions on the free movement of people by, for example, screening passengers at airports and refusing permission to travel, monitoring individuals’ movements by tracking mobile phone data or closing internal Union borders to people. For Union countries in the Schengen Area, in accordance with the Schengen Borders Code,^61^ Member States retain the power to check internal borders where public security is at issue,^62^ and to temporarily, and even immediately,^63^ reintroduce internal border controls where necessary because of a serious threat to “public policy or internal security”,^64^ including “contagious disease”.^65^ The Council has the power to recommend (but not mandate) such a reintroduction of internal border control.^66^

But, again, the web of competence would give the Union a basis to go further than recommending, as the Commission has already done,^67^ checks on people crossing internal borders.^68^ It would allow the Union to mandate such checks and to harmonise the conditions under which they are carried out.^69^

Taking matters even further by adopting a very innovative reading, the web of health competence could potentially even allow the Union to reinterpret the “no-bailout clause”,^70^ if it could be considered as part of the Union’s health powers (within a web of competence) in emergency circumstances, which would potentially open the way to the proposed “coronabonds” approach to the Union’s post-COVID-19 economic recovery.

## IV. Conclusion

Taken as a whole, the Union’s competences to respond to COVID-19 are wide ranging: much more so than traditional legal analysis suggests. The Union has powers that permit more actions than it has taken to date. Union competences, properly understood, support many of the actions the Commission has proposed and executed, *and* more. The Union’s competences also come with implicit responsibilities: if the Union has the power to respond to COVID-19, its citizens might legitimately expect it to do so. Further, with the responsibility to act come the implied means to take action: responsibility without enabling power and means (including funding) is meaningless. The web of competence is a legal basis to leverage that enabling power. The *legal* limitations on Union action are not the issue. Questions as to which actions to take, and whether there is a *political* or *policy desire* to take them, we leave to politicians, assisted by advisors such as virologists, behavioural psychologists, economists and epidemiologists.

## Footnotes

1 <https://euobserver.com/opinion/147841?utm_source=euobs&utm_medium=email> (last accessed 24 March 2020).

2 M Johnson, S Fleming and G Chazan, “Coronavirus: Is Europe Losing Italy?” *Financial Times* (6 April 2020) <https://www.ft.com/content/f21cf708-759e-11ea-ad98-044200cb277f> (last accessed 6 April 2020).

3 See KP Purnhagen, A de Ruijter, ML Flear, TK Hervey and A Herwig, “More Competences than You Knew”, work in progress. See, relatedly: A de Ruijter , EU Health Law and Policy: The Expansion of EU Power in Public Health and Health Care (Oxford, Oxford University Press 2019 ); A de Ruijter , “Mixing EU Security and Public Health in the Health Threats Decision” in A de Ruijter and M Weimer (eds), EU Risk Regulation, Expert and Executive Powe *r* (Oxford, Hart 2017 ) pp 101–120; ML Flear , Governing Public Health: EU Law, Regulation and Biopolitics (Oxford, Hart 2015, 2018 ); TK Hervey and JV McHale , European Union Health Law: Themes and Implications (Cambridge, Cambridge University Press 2015 ); TK Hervey and JV McHale , Health Law and the European Union (Cambridge, Cambridge University Press 2004 ).

4 Art 5 (2) sentence 1 TEU.

5 For discussion of the usual understanding of the Union’s competence norms, see, eg, P Craig , “EU Competences” in D Patterson and A Soederson (eds), A Companion to European Union Law and International Law (Hoboken, NJ, Wiley-Blackwell 2016) pp 83–94 .

6 Art 168 TFEU.

7 Art 196 TFEU.

8 European Commission, *Guidelines on EU Emergency Assistance on Cross-Border Cooperation in Healthcare related to the COVID-19 crisis* COM(2020) 2153 OJ C111I/1, 3 April 2020.

9 Arts 180 and 182 TFEU.

10 Arts 122 and 222 TFEU.

11 See, eg, European Parliament and Council Decision (EC) 2119/98 setting up a network for the epidemiological surveillance and control of communicable diseases in the Community OJ L 268/1; European Parliament and Council Decision (EU) 1082/2013 on serious cross-border threats to health and repealing Decision No 2119/98/EC OJ L 293/1.

12 This Committee, although in existence as inter-governmental cooperation before this date, was established as a Union Committee by European Parliament and Council Decision 1082/2013 (EU) on serious cross-border threats to health OJ L 293/1, Art 17. It is a formal Committee in the sense of European Parliament and Council Regulation (EU) 182/2011 laying down the rules and general principles concerning mechanisms for control by Member States of the Commission’s exercise of implementing powers OJ L 55/13, and it takes decisions in accordance with the “examination procedure” under Art 5 of that Regulation.

13 Such measures might require a dual legal basis of Art 168 TFEU and Art 80 (2) TFEU, the competence norm on which the European Parliament and Council Regulation (EC) 300/2008 on common rules in the field of civil aviation security OJ L 97/72 is based.

14 See European Commission, Proposal for a Regulation of the European Parliament and of the Council amending Regulation (EU) No 1303/2013, Regulation (EU) No 1301/2013 and Regulation (EU) No 508/2014 as regards specific measures to mobilise investments in the healthcare systems of the Member States and in other sectors of their economies in response to the COVID-19 outbreak [Coronavirus Response Investment Initiative] COM(2020) 113 final.

15 Arts 180 and 182 TFEU.

16 Art 222 TFEU. The “solidarity principle” enables the Union also to provide “measures appropriate to the economic situation, in particular if severe difficulties arise in the supply of certain products” (Art 122 (1) TFEU). The next paragraph of the same provision also enables the Council to provide financial assistance to a Member State “if [the Member State is in] difficulties or is seriously threatened with severe difficulties caused by natural disasters or exceptional occurrences beyond its control” (Art 122 (2) TFEU).

17 Council Decision (EU) 2014/415 on the arrangements for the implementation by the Union of the solidarity clause OJ L 192/53.

18 European Parliament and Council Decision (EU) 2019/420 on a Union Civil Protection Mechanism OJ L 77I/1.

19

JM Haussig et al, “The European Medical Corps: First Public Health Team Mission and Future Perspectives” (2017) 22(37) Eurosurveillance 30613 2893334310.2807/1560-7917.ES.2017.22.37.30613PMC5607656; European Commission Press Release: EU Launches New European Medical Corps to Respond Faster to Emergencies (MEMO/16/276), para 20; European Union Emergency Support Instrument for the healthcare sector – questions and answers 2 April 2020 <https://ec.europa.eu/commission/presscorner/detail/en/QANDA_20_577> (last accessed 4 April 2020).

20 Art 107(3)(c) TFEU; Art 107 (2)(b) TFEU. European Commission Communication, *Guidelines on State aid for rescuing and restructuring non-financial undertakings in difficulty* (2014) OJ C 249/1.

21 European Commission Communication, *Temporary Framework for State aid measures to support the economy in the current COVID-19 outbreak*, 20 March 2020, COM (2020) 1863 OJ C 91I/1; amended European Commission Communication, *Amendment to the Temporary Framework for State aid measures to support the economy in the current COVID-19 outbreak*, 3 April 2020, COM (2020) 2215 final <https://ec.europa.eu/competition/state_aid/what_is_new/sa_covid19_1st_amendment_temporary_framework_en.pdf>.

22 Communication from the Commission to the European Parliament, the European Council, the Council, the European Central Bank, the European Investment Bank and the Eurogroup, *Coordinated economic response to the COVID-19 Outbreak*, COM(2020) 112 final, 13 March 2020, Annex 3, p 6 <https://ec.europa.eu/info/sites/info/files/communication-annex-coordinated-economic-response-covid19-march-2020_en.pdf>. For example, a Brexit-preparedness measure for Ireland has been repurposed to help Irish firms cope with COVID-19, providing a €400 million support scheme for the acute liquidity and restructuring needs of small and medium-sized enterprises.

23 By, for instance, deferring payment of taxes and social security contributions by industries that have been badly affected (eg travel and tourism).

24 For example, medicinal products (including vaccines) and treatments, their intermediates, active pharmaceutical ingredients and raw materials; medical devices, hospital and medical equipment (including ventilators and protective clothing and equipment as well as diagnostic tools); disinfectants and their intermediary products and raw chemical materials necessary for their production and data collection and processing tools.

25 Art 114 TFEU; Art 168 (7) TFEU.

26 Art 114 TFEU; Art 168 (5) TFEU.

27 Case C-376/98 *Federal Republic of Germany v European Parliament and Council of the European Union (Tobacco Advertising)*, EU:C:2000:544, para 78.

28 Art 114 TFEU; Case C-376/98 *Federal Republic of Germany v European Parliament and Council of the European Union (Tobacco Advertising)*, EU:C:2000:544.

29 Art 114 (3) (4) TFEU.

30 Art 114 (8) TFEU; Case C-491/01 *British American Tobacco*, ECLI:EU:C:2002:741, para 76.

31 For example on the packaging of foods, European Parliament and Council Regulation (EC) 1935/2004 on materials and articles intended to come into contact with food and repealing, OJ L 338/4; and for other measures of the hygienic packaging for foodstuffs, see for a summary the Commission website <https://ec.europa.eu/food/safety/biosafety/food_hygiene/legislation_en>.

32 Arts 169 and 114 TFEU.

33 Arts 191 (1) and 192 TFEU.

34 WHO team, Department of Communications, Water, sanitation, hygiene and waste management for COVID-19 – Technical brief (19 March 2020) <https://www.who.int/publications-detail/water-sanitation-hygiene-and-waste-management-for-covid-19>.

35 The European Commission has proposed the delaying of the entry into force by one year of the European Parliament and Council Regulation on medical devices (EU) 2017/745 OJ L 117/1 due to the COVID-19 pandemic. The Directive is planned to take effect on 26 May 2020. The proposal aims to ensure the “smooth functioning of the internal market”, to provide a significant level of protection of public health and patient safety and to provide legal certainty and avoid market disruption; see Proposal for a Regulation of the European Parliament and Council amending Regulation (EU) 2017/745 on medical devices as regards the dates of application of certain of its provisions COM(2020) 144 final.

36 Arts 53(1) and 114 TFEU.

37 European Parliament and Council Directive (EC) 2005/36 on the recognition of professional qualifications OJ L 255/22, Art 25 (5), Annex V.

38 Art 168 (5) TFEU.

39 For example, Member States’ competent authorities and healthcare professionals have access to the COVID-19 Clinical Management Support System (CMSS) in order to ensure a quick exchange of knowledge and experience between clinicians from across the Union and European Economic Area on how to manage patients with severe COVID-19. Any clinician working in a hospital treating complex COVID-19 cases can have access to the web conferencing system and be supported by a dedicated helpdesk. See Guidelines on EU Emergency Assistance on Cross-Border Cooperation in Healthcare related to the COVID-19 crisis COM (2020) 111, 3 April 2020, para 8. Or the European Union’s Artificial Intelligence Robotics repository for sharing artificial intelligence/robotics solutions to COVID-19, see <https://ec.europa.eu/digital-single-market/en/news/join-ai-robotics-vs-covid-19-initiative-european-ai-alliance>.

40 European Parliament and Council Decision (EU) 1082/2013 on serious cross-border threats to health OJ L 293/1; Commission Implementing Decision (EU) 2017/253 laying down procedures for the notification of alerts as part of the early warning and response system established in relation to serious cross-border threats to health and for the information exchange, consultation and coordination of responses to such threats pursuant to Decision No 1082/2013/EU of the European Parliament and of the Council OJ L 37/23.

41 Which would be prohibited by Art 168 (7) TFEU.

42 Such as the long-standing cross-border health initiatives between France and Spain in the Cerdagne region or in the border region between Austria, the Czech Republic, Slovakia and Hungary, or Luxembourg Lorraine Santé, in the border region between France, Belgium and Luxembourg. These types of incentive measures are envisaged by the European Commission Guidelines on EU Emergency Assistance on Cross-Border Cooperation in Healthcare related to the COVID-19 crisis COM(2020) 2153 OJ C111I/1, 3 April 2020.

43 See, eg, Case 8/74 *Dassonville* ECLI:EU:C:1974:82 (“direct or indirect, actual or potential”); Case C-60/00 *Carpenter* ECLI:EU:C:2002:434, where the potential effect on cross-border service provision was enough.

44 Case C-183/95 *Affish BV v Rijksdienst voor de Keuring van Vee an Vlees* ECLI:EU:C:1997:373, para 43; Case C-221/10-P *Artegodan GmbH v European Commission*, ECLI:EU:C:2012:216, para 99.

45 Arts 9, 168 (1) TFEU, Art 35 EU CFR.

46 Art 352 TFEU.

47 The Treaty of Maastricht, which came into effect on 1 January 1993, included what is now Art 168 TFEU for the first time.

48 See KP Purnhagen , The Politics of Systematization in EU Product Safety Regulation (Springer 2013 ) at pp 184–85; de Ruijter, *EU Health Law and Policy*, supra, note 3, 57.

49 COM(2020) 112 final, supra, note 22. Note, substances of human origin are also “essential products”, see DG Sante Note <https://ec.europa.eu/health/sites/health/files/blood_tissues_organs/docs/2020_soho_crossbordershipments_en.pdf>.

50 Art 168 TFEU.

51 Duly following procedural obligations in Art 114 (4)–(8) TFEU.

52 Case C-333/14 *Scotch Whisky Association v The Lord Advocate of Scotland* ECLI:EU:C:2015:845, paras 34, 38.

53 For a requirement to adopt new steering principles to coordinate diversity in the EU, which are different from the conventional tools such as proportionality, subsidiarity and conferral, see KP Purnhagen , “From Supranationality to Managing Diversity – A (Re-)New(ed) Paradigm for the Establishment of the Internal Market?” in I Govaere and S Garben (eds), Internal Market 2.0 (Oxford, Hart 2020 , in press).

54 Arts 122 and 222 TFEU.

55 Art 9 TFEU.

56 Art 35 EU CFR.

57 COM(2020) 113 final, supra, note 22, Annex 2, p 4.

58 COM(2020) 112 final, supra, note 22, Annex 2, p 4: “The recent decisions by Member States to ban or restrict exports – in one case extending to of [sic] 1324 products, including paracetamols and medical devices – contribute to the risk of shortages in other Member States, thereby putting at risk the health of people living in Europe *and should be corrected as a matter of urgency*” (emphasis added); p 4: “A simple export ban alone cannot meet the legal requirement of proportionality”. However, other measures that may create barriers to free movement, such as price regulation in Member States, as long as these are not discriminatory, are allowed according to the Commission COM(2020) 112 final, supra, note 22, Annex 2, p 5, as are other national measures to regulate the market of medical supplies.

59 Incidentally, the work done by the Commission (and UK) on the effects of a “No Deal” Brexit has helped to highlight the nature of European supply chains for essential products, especially food and medicines/devices/equipment.

60 For movement *within* the Union, according to Arts 45 (3), 52 and 62 TFEU, and various provisions in long-standing Union legislation, eg, Directive 2004/38 (citizens) Arts 27–33; Directive 2003/109 (long-term residents), Arts 17–18. For movement *into* the Union, Art 77 (2) (b), (c), (d) TFEU; Art 79 (2) (a) TFEU, European Parliament and Council Regulation (EU) 2016/399 on a Union Code on the rules governing the movement of persons across borders (Schengen Borders Code) OJ L 77/1; European Commission, *COVID-19 Guidelines for Border Management Measures to Protect Health and Ensure the Availability of Goods and Essential Services*, 16 March 2020, COM(2020) 1753 final.

61 Schengen Borders Code, supra, note 60.

62 Schengen Borders Code, supra, note 60, Art 23 (a) (ii), (b).

63 Schengen Borders Code, supra, note 60, Art 28.

64 Schengen Borders Code, supra, note 60, Arts 25–27.

65 COM(2020) 1753 final, supra, note 60.

66 Schengen Borders Code, supra, note 60, Arts 29–30.

67 COM(2020) 1753 final, supra, note 60.

68 According to the guideline, permissible health checks can be carried out by border patrols (primary screening) or by medical staff (secondary screening). The checks should be limited to health checks on people crossing internal borders and do not imply the reintroduction of border controls generally, especially not on goods. The guideline recommends that, if suspected cases need immediate isolation and/or medical treatment, the authorities on each side of the border should coordinate where quarantine and treatment is to take place; see COM(2020) 1753 final, supra, note 60, paras 11 and 19.

69 For instance, to secure compliance with human rights protection and non-discrimination on forbidden grounds, as well as to be organised in a way so as to avoid queues and large gatherings so as to limit the spread of COVID-19.

70 Art 125 (1) TFEU.

